# Ethanol fuel improves arthropod capture in pitfall traps and preserves DNA

**DOI:** 10.3897/zookeys.196.3130

**Published:** 2012-05-21

**Authors:** Neucir Szinwelski, Verônica S. Fialho, Karla S. C. Yotoko, Léon R. Seleme, Carlos F. Sperber

**Affiliations:** 1 Postgraduate Programme in Entomology, Department of Entomology, Federal University of Viçosa, Avenida P.H. Rolfs s/n, Centro, Viçosa, Minas Gerais, Brazil; 2 Laboratory of Orthoptera, Federal University of Viçosa, Avenida P.H. Rolfs s/n, Centro, Viçosa, Minas Gerais, Brazil; 3 Laboratory of Bioinformatics and Evolution, Federal University of Viçosa, Avenida P.H. Rolfs s/n, Centro, Viçosa, Minas Gerais, Brazil

**Keywords:** Killing solutions, Molecular tools, Taxonomy, Large-scale fieldwork, Brazil

## Abstract

We tested the value of ethanol fuel as a killing solution in terms of sampling efficiency (species richness and accumulated abundance) and DNA preservation of Ensifera ground-dwelling specimens. Sampling efficiency was evaluated comparing abundance and species richness of pitfall sampling using 100% ethanol fuel, with two alternative killing solutions. We evaluated the DNA preservation efficiency of the killing solutions and of alternative storage solutions. Ethanol fuel was the most efficient killing solution, and allowed successful DNA preservation. This solution is cheaper than other preserving liquids, and is easily acquired near field study sites since it is available at every fuel station in Brazil and at an increasing number of fuel stations in the U.S. We recommend the use of ethanol fuel as a killing and storage solution, because it is a cheap and efficient alternative for large-scale arthropod sampling, both logistically and for DNA preservation. For open habitat sampling with high day temperatures, we recommend doubling the solution volume to cope with high evaporation, increasing its efficacy over two days.

## Introduction

Several sampling techniques are used to assess biodiversity of different animal species (King and Porter 2005). All present advantages and disadvantages, so the choice is at the discretion of the researcher. Small organisms (e.g. arthropods) are frequently hand-sampled, which provides information on the organism’s habits and behavior, but this method is of little use for ecological comparisons, because of collector interference (Krebs 1999, Southwood and Henderson 2000).

Pitfall traps are a good alternative for collecting ground-dwelling arthropods (Dahl 1896). This kind of trap is inexpensive and easy to handle, allowing both rich and abundant samples. It can be used for taxonomic (although some coloration characters may be lost), ecological, morphological and molecular studies (Gurdebeke and Maelfait 2002, Schoereder et al. 2004, Sperber et al. 2007, Mews et al. 2008, Pereira et al. 2010). One of the main challenges is deciding which killing solution to use in the pitfall traps, which depends on the objectives of each study. As far as sampling involves financial, environmental and researcher's effort costs, the ideal solution should minimize those costs and maximize the utility of the sampled material. The utility of the samples may extrapolate strictly ecological purposes, and should involve other scientific areas, such as morphology and molecular biology. Therefore an ideal should also preserve the specimens' tissues and DNA (Stevens et al. 2011).

Regarding methodological necessities in pitfall sampling, a good killing solution should minimize evaporation, as far as many pitfall trap regimes check traps every 2 weeks or more. A good solution should not be toxic to the researcher nor environmentally harmful. Regarding sampling efficiency, a good solution should kill quickly so as to reduce the escape of specimens. In addition, the trap solution cannot be prohibitively expensive, and must be readily available.

Finding a solution that meets all of these specifications is not easy. Many types of solutions have been used and tested, for example water and detergent, which is inexpensive but accelerates the decomposition of tissues and genetic material (Schmidt et al. 2006). Mixtures of formaldehyde and ethylene glycol (Barber 1931, Sperber et al. 2003b, Schmidt et al. 2006), are efficient in killing and preserving tissue, but are toxic and do not preserve DNA (Aristophanous 2010). Other solutions contain salt brines (Sasakawa 2007) and acetic acid (Gurdebeke and Maelfait 2002), which do not preserve tissues and can alter gonads, genitalia and eggs (Sasakawa 2007). An additional class of solutions contains different concentrations of commercial alcohol (Sperber et al. 2003a, Paquin 2008, Chen et al. 2011), which evaporates faster than the other solutions, but preserves the internal and external organs through tissue dehydration.

It has been shown that at concentrations higher than 95%, commercial alcohol preserves DNA (Nagy 2010), but the use of highly concentrated commercial alcohol as a killing solution may be prohibitively expensive when needed in large quantities, such as in large-scale biodiversity sampling. In Brazil, for example, it is illegal to carry large amounts of commercial alcohol on long journeys, which could hinder its use in extensive field expeditions. Here we propose the use of ethanol fuel as a cheaper and logistically feasible alternative.

In Brazil, ethanol fuel and commercial alcohol have some differences. While the alcoholic concentration (92.6 to 93.8%) and the amount of water (6.2 to 7.4%) varies in ethanol fuel, in commercial alcohol the alcoholic concentration (92.8%) and the amount of water (7.2%) is fixed. The largest difference is, however, the quantity of gasoline present in ethanol fuel (up to 30 milliliters per liter), that is absent in commercial alcohol (BR0029 2011). In the United States, the highest concentration of ethanol fuel includes 85% ethanol and 15% gasoline (Tatum 2010). Ethanol fuel is available throughout Brazil, at all fuel stations, and at an increasing number of fuel stations in the U.S. (Méjean and Hope 2010, Sorda et al. 2010) and is at least 50% cheaper than commercial alcohol.

In this study, we tested the value of ethanol fuel as a pitfall trap killing solution in terms of sampling efficiency (richness and abundance) and DNA preservation of Ensifera ground-dwelling specimens, comparing 100% ethanol fuel with two alternative killing solutions.

## Material and methods

### Sampling efficiency

Field sampling site

To evaluate sampling efficiency, we conducted field sampling in a primary Atlantic Forest reservoir, the Iguaçu National Park, in Foz do Iguaçu municipality (25°32'S, 54°35'W, 195 m above sea level), Paraná State, in January 2010. The vegetation is mostly tropical semideciduous forest and Araucaria forest, within the Atlantic Forest biome (Rizzini 1997, Dias et al. 1998). The climate is mesothermal subtropical superhumid, with average annual temperatures between 18 and 20 °C and an average rainfall of 1600mm (Peel et al. 2007).

Sampling design

We compared the efficiency of 100% ethanol fuel pitfall killing solution (Solution 1) for ground-dwelling Orthoptera, against the conventional killing solution, comprised of 80% commercial alcohol (80°GL) + 10% glycerin (P.A) + 10% formaldehyde (P.A) (Sperber et al. 2003b) (Solution 2), and a solution of 90% commercial alcohol (80°GL) + 10% glycerin (P.A) (Solution 3). GL is the amount, in milliliters, of absolute alcohol contained in 100 milliliters of hydro-alcoholic solution. P.A., or ‘Pro Analysis’ means that the sample is of a very high purity, sufficient to be used in chemical analyses. Formaldehyde is recommended for better preservation; glycerin is used to prevent stiffening of the sampled specimens.

For this comparison, we designed the following field experiment. We established a transect of 5km, starting at a distance of 100m from the forest’s edge. At the beginning of the transect a set of five pitfall traps, containing one of the three killing solutions chosen randomly, were placed perpendicularly to the transect, 2m apart from one another. After the next 30m on the transect, we placed the second set with a different, randomly chosen, killing solution. After another 30m along the transect, we placed the third set, with the third killing solution. After an additional forty meters we began the procedure again, and repeated it a total of 50 sampling stations. In summary each sampling station contained five pitfall traps with each of the three killing solutions, for a total sampling effort of 750 pitfall traps. Traps consisted of polyethylene vials, 20cm in diameter and 22cm deep, filled with 500ml of killing solution. After 48 hours, specimens were removed from the the traps, identified and stored in ethanol fuel, after gathering the data.

Data analysis

To evaluate sampling efficiency of ethanol fuel as a pitfall killing solution, we compared cricket species richness and accumulated abundance (= total number of individuals per pitfall set) among the three solutions. Each pitfall set was considered one sampling unit, rendering 150 replicates. We performed one-way analysis of variance (ANOVA), adjusting generalized linear models (GLMs) with Poisson error distribution, correcting for over- or under-dispersion using quasi-Poisson when necessary. We considered cricket species richness and accumulated abundance in each set of five pitfall traps as response variables (n = 150), and the type of killing solution as the explanatory factor. We used contrast analyses to evaluate effect differences among the kinds of solution, simplifying the complete models by amalgamating non-significantly different factor levels (Crawley 2007). We used Chi-square (χ^2^) test for Poisson error distributions, and the *F* test in cases where there was a correction for over- or under-dispersion, as recommended by Zuur et al. (2009). We checked residuals for homoscedasticity. All analyses were undertaken within the R 2.15 environment (R Development Core Team 2012).

### DNA preservation

Killing and storage

To test the DNA preservation properties of each pitfall killing solution, we placed each of 18 living cricket specimens of *Gryllus* sp. (not identified) into one of the three pitfall killing solutions, totaling six specimens per solution. As a control, we separately placed another six crickets into undiluted commercial alcohol (92.8°GL), which is considered a good preservative of DNA (Nagy 2010). Twenty-four hours later, we took one leg of each individual and extracted its DNA. Twenty-four hours later (*i.e*. 48 hours after immersion into the killing solution), we removed a second leg off the crickets to evaluate DNA preservation, analogous to in the field procedure collecting time of 48 hours, as recommended by Sperber et al. (2003a) for ground-dwelling Orthoptera sampling.

To evaluate the efficiency of ethanol fuel as a storage solution, we stored each cricket specimen, after 48 hours in the killing solution, in one of two storage solutions: undiluted commercial alcohol (92.8°GL) or undiluted ethanol fuel. To test the effect of time and type of storage solution on the DNA preservation efficiency, we removed a third leg off each cricket after 15 days, and a fourth leg after 30 days in the storage solution.

We evaluated efficiency of DNA preservation for the 24 crickets used in the above procedure. Each set of six individuals was submitted to one of four different killing solutions, and each individual provided two samples (= legs) for DNA extraction before storage (24 and 48 hours in the killing solution). Individuals from each killing solution were transferred to either commercial alcohol or ethanol fuel for storage, providing three replicates (individuals) per storage solution, and two further samples (= legs) per individual, 15 and 30 days in the storage solution. All specimens were maintained at room temperature for 30 days.

DNA extraction

Total DNA was isolated from each individual using the protocol described in Waldschmidt et al. (1997) but without the deproteinization step with phenol:chloroform (1:1). Preliminary analysis of fresh specimens killed by freezing showed that tissue extractions from the thorax or legs were equally effective. Therefore, we chose to use only the legs, allowing maximum preservation of anatomical parts for further studies, and repeated sampling of the same individuals with minimum tissue damage.

DNA extractions were verified via agarose gel (0.8%) electrophorese, prepared and run in 1X TBE Buffer, stained with ethidium bromide and viewed under UV light. The quality of the extractions was checked by comparison with the extract made from fresh material (specimens that were killed by freezing, with immediate DNA extraction). Extractions from fresh material presented two bands, the first clearly marked and bright, corresponding to genomic DNA and the second smaller, more opaque, corresponding to RNA. We considered DNA as properly preserved when we detected a well-defined single band of DNA without apparent trawlers.

## Results

### Sampling efficiency

**Figure 1.**
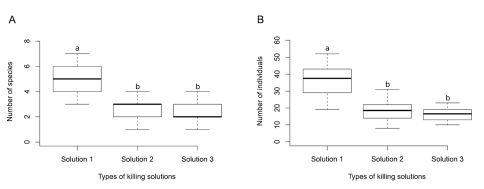
Boxplot showing sampling efficiency of different kinds of pitfall traps' killing solution. Traps with **Solution 1** (100% ethanol fuel) captured more species and individuals than **Solution 2** (80% commercial alcohol (80°GL) + 10% glycerin (P.A) + 10% formaldehyde (P.A)) and **Solution** **3** (90% commercial alcohol (80°GL) + 10% glycerin (P.A)). **A** Total number of species per pitfalls’ set. **B** Total number of individuals per pitfalls’ set. Different lower case letters correspond to significant differences between killing solution levels, evaluated through contrast analyses.

We collected 3,528 individuals of 14 species from four different families of Orthoptera, following the classification of Desutter-Grandcolas (1987, 1988): Phalangopsidae (2,090 individuals of eight species), Trigonidiidae (835 individuals of two species), Gryllidae (394 individuals of two species) and Eneopteridae (209 individuals of two species). Species richness (F_2,147_ = 177.09; p < 0.001) and abundance (F_2,147_ = 104.64; p < 0.001) were significantly higher in pitfalls with ethanol fuel killing solution (Figure 1 A, B) than in those containing the other two solutions. Sampling efficiency was not different between killing solution 2 and 3 (richness: F_2,147_ =0.34; p = 0.55; abundance: F_2,147_ = 2.87; p = 0.09).

### DNA Preservation

**Figure 2.**
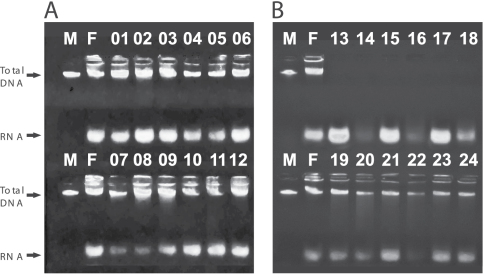
Electrophoresis of all 24 analyzed individuals. M represents the lambda DNA marker (100 ng/ul) and F represents the control extraction made using fresh tissue. A) Lanes 01 – 06, individuals killed in C.A. (undiluted commercial alcohol), maintained in the killing solution for 48 hours and then transferred to closed vials containing C.A. (01 – 03) and E.F. (03 – 06) and maintained in these storage solutions for 30 days. Lanes 07 – 12, individuals killed in Solution 1 (= E.F.), maintained in the killing solution for 48 hours and transferred to C.A. (07 – 09) and E.F. (10 – 12) and maintained in these storage solutions for 30 days. B) Lanes 13 – 18, individuals killed in the Solution 2 and maintained in this solution for 24 hours. Lanes 19 – 24, individuals killed in Solution 3, maintained in this solution for 48 hours, than transferred to C.A. (19 – 21) and E.F. (22 – 24) and maintained in these solutions for 30 days. All DNA extractions where successful, but those of crickets killed in solution 2 (lanes 13 – 18).

Table 1 indicates that both solution 1 and solution 3 were efficient in preserving DNA and are appropriate for use as killing solutions in pitfall traps that must remain in the field for up to 48 hours, with no visible damage to DNA. In addition, these samples can be stored at room temperature for up to 30 days in either commercial alcohol or ethanol fuel. On the other hand, our results suggest that just 24 hours in solution 2 (commercial alcohol + glycerin + formaldehyde) are enough to destroy the DNA of the samples (Figure 2).

**Table 1.** Success (yes) or failure (no) of DNA extractions after different periods (Time in the solution) in Killing solution (Pitfall: 24h and 48h) and in storage solution (C.A. and E.F.: 15 and 30 days). C.A. = undiluted commercial alcohol (92.8°GL); E.F. = undiluted ethanol fuel; Solution 1 = E.F.; Solution 2 = 80% commercial alcohol (80°GL) + 10% glycerin (P.A.) + 10% formaldehyde (P.A.); Solution 3 = 90% commercial alcohol (80°GL) + 10% glycerin (P.A.). All material was maintained at room temperature. Asterisks mark the treatments shown in Figure 2.

**Killing solutions**	**Time in the solution**					
	Pitfall		C.A.		E.F.	
	24h	48h	15days	30days	15days	30days
C.A.	yes	yes	yes	yes*	yes	yes*
Solution 1	yes	yes	yes	yes*	yes	yes*
Solution 2	no*	-	-	-	-	-
Solution 3	yes	yes	yes	yes*	yes	yes*

## Discussion

In this study, we investigated the efficiency of ethanol fuel as a pitfall killing solution in terms sampling efficiency, as measured by species richness and accumulated abundance, and in terms of DNA preservation. Our results indicate increased sampling and preservation efficiency of ethanol fuel, compared to the commonly used alternatives. Below we discuss the advantages and disadvantages of using ethanol fuel as a pitfall killing and storage solution, with particular emphasis on large-scale field expeditions.

### Financial costs

Of the solutions tested in our study, ethanol fuel is the least expensive option: 1 liter of ethanol fuel (US$ 1.25 on average) costs less than half the price of 1 liter of commercial alcohol (US$ 3.15), which does not include the other components, such as glycerin and formaldehyde, which cost around US$ 15.00 a liter (prices for Brazil).

### Field logistics

The transportation of flammable or toxic liquids is dangerous and illegal under Brazilian and international law. This danger increases with the distance, and consequently time spent in transportation. Ethanol fuel presents a partial solution to this limitation: as it can be bought near the field study sites, at any fuel station in Brazil, the distance of transportation is diminished, decreasing the danger. Large field expeditions can use these facilities to reduce the distances of ethanol transportation, thus reducing the risks of accidents, and simplifying expedition logistics. Even so, for transportations and storage of collected material, we recommend using firm, pressure-resistant bottles, with sealed caps, fully filled with ethanol, so as to minimize oxygen within the bottle, reducing explosion risks. We used PET tubes, which have low costs and may be bought in large quantities.

Commercial alcohol has to be purchased in large shops when bought in large quantities, and is hardly available in the small towns that border most of the large conservation areas. Therefore it would require long-distance transportation and represent huge environmental and personal risks. The additional components of the tested killing solutions (glycerin and formaldehyde), are only available in specialized establishments, restricted to a few large cities in Brazil (Brazilian Federal Law n°10.357/2001).

### Sampling efficiency

We showed that ethanol fuel presented higher sampling efficiency, both for species richness and accumulated abundance of ground-dwelling Orthoptera species, therefore maximizing the gains of the sampling effort. We hypothesize that this higher sampling efficiency is related to the lower density and surface tension of the solution 1 (density = 0.81 g/cm^3^; surface tension = 21.55 mN/m^-1^) than solution 2 (density = 0.92 g/cm^3^; surface tension = 48.56 mN/m^-1^ ) and solution 3 (density = 0.97 g/cm^3^; surface tension = 55.34 mN/m^-1^) (Adamson and Gast 1997), which could cause the crickets to sink and die faster in ethanol fuel, reducing their chances of escape from the trap.

One piece of evidence in favor of our hypothesis is that all winged cricket species captured in this study died exclusively within pitfalls that used ethanol fuel as the killing solution (94 individuals of *Eneoptera* sp. and 183 individuals of *Gryllus* sp.). These genera contain species of large body size, which are powerful jumpers as nymphs and powerful fliers as adults, and are rarely captured in conventional pitfall traps killing solution (N. Szinwelski, personal observation). Indeed, C.F. Sperber, in other field collections, has observed adults of *Eneoptera* sp. flying out of pitfalls with water + detergent killing solution. The alternative pitfall design used to prevent escape from traps, using an inverted funnel at the trap’s top (Melbourne et al. 1997), may reduce sampling efficiency, especially for good jumpers and fliers.

### DNA preservation efficiency

To obtain DNA samples, it is recommended that the sampled organisms be removed from the pitfall killing solution as soon as possible and placed in vials containing highly concentrated alcohol, preferably at low temperatures (Nagy 2010). Based on the results presented here, we suggest that sampled organisms may be safely stored in undiluted ethanol fuel at room temperature, without major damage to DNA quality, for up to 30 days.

Indeed, we were able to obtain sequences of mitochondrial DNA (COI) and nuclear (18S rRNA) of Orthoptera specimens kept for two weeks in ethanol fuel killing solution, before being sorted and stored in undiluted commercial ethanol (92.8°GL), where they remained at 38°C – 45°C room temperature for another 45 days (in Manaus – AM) and 70 days at similar temperature (in Cuiabá – MT).

### Counterarguments

One of the main arguments against the use of ethanol fuel as a pitfall trap killing solution is that it evaporates faster than other solutions, making its use limited to high temperature areas. We were, however, able to use ethanol fuel pitfall traps successfully in Amazon forest sampling (38°C – 45°C), where the traps were kept for 48h in the field without significant volume reduction of the killing solution.

Solution evaporation is a limiting factor in open habitat with high temperatures as Brazilian “Campo Cerrado”, for example. In such field conditions, we recommend increasing the killing solution volume by 100%, from 500ml to 1000ml, to maintain sufficient killing solution volume in the traps after 48h in the field.

Another problem with ethanol fuel is the fact that it can be denatured. In Brazil, that means that every liter of ethanol fuel can contain up to 30ml of gasoline. In the United States every liter of ethanol E85 contain 150ml of gasoline. This may represent an environmental problem if the pitfall is damaged and the solution is spread in the environment. Moreover, gasoline might hinder DNA preservation. For Brazilian ethanol fuel we showed that this did not occur. Even specimens collected in ethanol fuel, were successfully preserved and we were able to extract DNA and run PCR reactions obtaining sequences of mitochondrial COI and nuclear rRNA18S .

## References

[B1] Adamson AW Gast AP (1997) Physical chemistry of surfaces, 6th edition. Wiley-Interscience, New York.

[B2] Aristophanous M (2010) Does your preservative preserve? A comparison of the efficacy of some pitfall trap solutions in preserving the internal reproductive organs of dung beetles. ZooKeys 34 (1): 1-16.

[B3] Barber HS (1931) Traps for cave-inhabiting insects. Journal of the Mitchell Society 46: 259-266.

[B4] Chen Y Li Q Wang S Zhou X (2011) A comparison of pitfall traps with different liquids for studying ground-dwelling ants (Hymenoptera: Formicidae). Myrmecological News 14 (1): 13-19.

[B5] Crawley MJ (2007) The R book. John Wiley & Sons, Ltd, West Sussex - UK. doi: 10.1002/9780470515075

[B6] Dahl F (1896) Vergleichende Untersuchungen über die Lebensweise wirbelloser Aasfresser. Sitzber Königl PreußAkad Wiss 1: 11-24.

[B7] Desutter L (1987) Structure et évolution du complexe phallique des Gryllidea (Orthoptères) et classification des genres néoropicaux de Grylloidea - primèire partie. Annales de la Société Entomologique de France 23 (3): 213-239.

[B8] Desutter L (1988) Structure et évolution du complexe phalique des Grylloidea (Orthoptères) et classification des genres néotropicaux de Grylloidea: deuxième partie. Annales de la Société Entomologique de France 24 (3): 343-373.

[B9] Dias MC Vieira AOS Nakajima JN Pimenta JA Lobo PC (1998) Composição florística e fitossociologia do componente arbóreo das florestas ciliares do rio Iapó, na bacia do rio Tibagi, Tibagi, PR. Revista Brasileira de Botânica 21: 183-195.

[B10] Gurdebeke S Maelfait JP (2002) Pitfall trapping in population genetics studies: Finding the right “solution”. The Journal of Arachnology 30 (1): 255-261. doi: 10.1636/0161-8202(2002)030[0255:PTIPGS]2.0.CO;2

[B11] King JR Porter SD (2005) Evaluation of sampling methods and species richness estimators for ants in upland ecosystems in Florida. Environmental Entomology 34 (6): 1566-1578. doi: 10.1603/0046-225X-34.6.1566

[B12] Krebs CJ (1999) Ecological methodology, 2nd edition. Addison-Wesley Educational Publishers, Inc.

[B13] Melbourne BA Gullan PJ Su YN (1997) Interpreting data from pitfall-trap surveys: Crickets and slugs in exotic native grasslands of the Australian capital territory. Memoirs of the Museum of Victoria 56 (2): 361-367.

[B14] Méjean A Hope C (2010) Modelling the costs of energy crops: A case study of US corn and Brazilian sugar cane. Energy Policy 38 (1): 547-561. doi: 10.1016/j.enpol.2009.10.006

[B15] Mews CM Lopes-Andrade C Sperber CF (2008) A new species of *Laranda* Walker 1869 (Orthoptera, Grylloidea, Phalangopsidae) from remnant patches of the Brazilian Atlantic Forest. Neotropical Entomology 37 (4): 420-425. doi: 10.1590/S1519-566X2008000400010 18813744

[B16] Nagy ZT (2010) A hands-on overview of tissue preservation methods for molecular genetic analyses. Organisms Diversity & Evolution 10 (1): 91-105. doi: 10.1007/s13127-010-0012-4

[B17] Paquin P (2008) Carabid beetle (Coleoptera: Carabidae) diversity in the black spruce succession of eastern Canada. Biological Conservation 141 (1): 261-275. doi: 10.1016/j.biocon.2007.10.001

[B18] Peel MC Finlayson BL McMahon TA (2007) Updated world map of the Köppen-Geiger climate classification. Hydrology Earth System Science 11: 1633-1644. doi: 10.5194/hess-11-1633-2007

[B19] Pereira MR Sperber CF Lhano MG (2010) First report and three new species of *Amanayara* (Orthoptera: Grylloidea) in Minas Gerais State, Brazil. Zootaxa 2542: 1-17.

[B20] R Development Core Team (2012) R: A language and environment for statistical computing. R Foundation for Statistical Computing, Viena - Austria, http://www.r-project.org

[B21] Rizzini CT (1997) Tratado de fitogeografia do Brasil: aspectos ecológicos, sociológicos e florísticos, 2nd ed. Âmbito Cultural, Rio de Janeiro.

[B22] Sasakawa K (2007) Effects of pitfall trap preservatives on specimen condition in Carabid beetles. Entomologia Experimentalis et Applicata 125 (3): 321-324. doi: 10.1111/j.1570-7458.2007.00620.x

[B23] Schmidt MH Clough Y Schulz W Westphalen A Tscharntke T (2006) Capture efficiency and preservation attributes of different fluids in pitfall traps. Journal of Arachnology 34 (1): 159-162. doi: 10.1636/T04-95.1

[B24] Schoereder JH Galbiati C Ribas CR Sobrinho TG Sperber CF DeSouza O Lopes-Andrade C (2004) Should we use proportional sampling for species-area studies? Journal of Biogeography 31 (8): 1219–1226. doi: 10.1111/j.1365-2699.2004.01113.x

[B25] Sorda G Banse M Kemfert C (2010) An overview of biofuel policies across the world. Energy Policy 38 (11): 6977-6988. doi: 10.1016/j.enpol.2010.06.066

[B26] Southwood TRE Henderson PA (2000) Ecological Methods. Wiley-Blackwell.

[B27] Sperber CF Rocha A Lopes-Andrade C Mesa A (2003a) *Izecksohniella puri* sp. n., a new Brazilian cricket species (Orthoptera: Grylloidea: Phalangopsidae) from Atlantic Forest remnants. Zootaxa 244: 1-12.

[B28] Sperber CF Vieira GH Mendes MH (2003b) Aprimoramento da amostragem de grilos de serapilheira (Orthoptera: Gryllidae) por armadilha. Neotropical Entomology 32 (4): 733-735. doi: 10.1590/S1519-566X2003000400030

[B29] Sperber CF Soares LGS Pereira MR (2007) Litter disturbance and trap spatial positioning affects number of captured individuals and genera of crickets (Orthoptera: Grylloidea). Journal of Orthoptera Research 16 (1): 77-83. doi: 10.1665/1082-6467(2007)16[77:LDATSP]2.0.CO;2

[B30] Stevens MM Warren GN Mo J Schlipalius DI (2011) Maintaining DNA quality in stored-grain beetles caught in Lindgren funnel traps. Journal of Stored Products Research 47 (2): 69-75. doi: 10.1016/j.jspr.2010.10.002

[B31] Tatum SW Skinner SJ Jackson JD (2010) On the economic sustainability of ethanol E85. Energy Economics 32 (1): 1263-1267. doi: 10.1016/j.eneco.2010.08.001

[B32] Waldschmidt AM Salomão TMF Barros EG Campos LDAO (1997) Extraction of genomic DNA from *Melipona quadrifasciata* (Hymenoptera: Apidae, Meliponinae). Brazilian Journal of Genetics 20 (3): 421-423. doi: 10.1590/S0100-84551997000300011

[B33] Zuur AF Ieno EN Walker NJ Saveliev AA Smith GM (2009) Mixed effects models and extensions in ecology with R. Springer Press, New York. doi: 10.1007/978-0-387-87458-6

